# Three-dimensional micro-X-ray topography using focused sheet-shaped X-ray beam

**DOI:** 10.1038/s41598-023-39347-4

**Published:** 2023-07-31

**Authors:** Akio Yoneyama, Kotaro Ishiji, Atsushi Sakaki, Yutaka Kobayashi, Masayuki Inaba, Kazunori Fukuda, Kumiko Konishi, Akio Shima, Daiko Takamatsu

**Affiliations:** 1 Research and Development Group, Hitachi Ltd., 1-280 Higashi-Koigakubo, Kokubunji, 185-8601 Japan; 2 SAGA Light Source, 8-7 Yayoigaoka, Tosu, 841-0005 Japan; 3 Phosphor R&D Center, Nichia Corporation, 1-19 Tatsumi, Anan, 774-0001 Japan; 4 Chip Development Department, Nichia Corporation, 491 Oka, Kaminaka, Anan 774-8601 Japan; 5 Nissan ARC, Ltd. 1 Natsushima-Cho, Yokosuka, 237-0061 Japan; 6 Physical Analysis Center, Kobelco Research Institute, Inc., 1-5-5 Takatsukadai, Nishi-Ku, Kobe, 651-2271 Japan; 7 Research and Development Group, Hitachi Ltd., 2520 Akanuma, Hatoyama, 350-0395 Japan

**Keywords:** Materials science, Materials for devices, Techniques and instrumentation, X-rays, Imaging techniques

## Abstract

X-ray topography is a powerful method for analyzing crystal defects and strain in crystalline materials non-destructively. However, conventional X-ray topography uses simple X-ray diffraction images, which means depth information on defects and dislocations cannot be obtained. We have therefor developed a novel three-dimensional micro-X-ray topography technique (3D μ-XRT) that combines Bragg-case section topography with focused sheet-shaped X-rays. The depth resolution of the 3D μ-XRT depends mainly on the focused X-ray beam size and enables non-destructive observation of internal defects and dislocations with an accuracy on the order of 1 μm. The demonstrative observation of SiC power device chips showed that stacking faults, threading screw, threading edge, and basal plane dislocations were clearly visualized three-dimensionally with a depth accuracy of 1.3 μm. 3D μ-XRT is a promising new approach for highly sensitive and non-destructive analysis of material crystallinity in a three-dimensional manner.

## Introduction

X-ray topography has been widely utilized for non-destructive and highly sensitive analysis of crystal distortions, dislocations, and defects in crystalline materials such as wafers, ingots, and semiconductor devices. However, depth information cannot be obtained because crystallinity information is generally obtained from the two-dimensional intensity distribution (topogram) of reflected or transmitted X-ray diffraction. Therefore, three-dimensional analysis of crystal defects and distortions cannot be performed generally, and it is impossible to determine whether a crystal defect is near the surface or deep within the material. Although stereographic observations have been performed and 3D defects within the crystals were obtained^1, 2^, it has yet to be possible to identify its depth on the micron order. Therefore, depth analysis of stacking faults in the epitaxial layer, which cause the degradation of semiconductor power devices, cannot be performed. Section topography, topo-tomography, and scanning micro-topography using a focused X-ray microbeam have been developed for the three-dimensional characterization of crystalline materials. Section topography^3, 4^ obtains a three-dimensional topogram by stacking multiple topograms obtained using sheet-shaped X-rays by scanning the sample. It has been used to visualize the three-dimensional structure of defects in the neck of a crystal ingot^5^, along with other applications. However, the spatial resolution depends mainly on the sheet height of the X-ray beam and is limited to the sub-mm order. A recently proposed micro-topography technique called dark-field X-ray microscopy (DFXM) that utilizes a focused sheet-shaped X-ray is reported to obtain a detailed three-dimensional distortion map in a bulk aluminum block^6^. However, the field of view was limited to 100 μm by the imaging X-ray lens, and observations were made only in the transmission geometry (Laue-case). Therefore, long measurement time is required to observe an entire power device of several mm squares by scanning.

The topo-tomography method^7^ is similar to X-ray computed tomography in that the sample is rotated and the three-dimensional distribution is calculated from the topogram obtained at each rotational angle. It has been combined with white synchrotron radiation (SR) to perform three-dimensional observation of dislocation propagations in the early stage of Czochralski silicon crystal growth^8^. However, the spatial resolution is mainly determined by the X-ray imager and the distance between the sample and the X-ray imager (working distance (WD)), as in the case of X-ray micro-CT with parallel beam geometry, and is about 10 μm at minimum. In addition, transmission geometry is generally used for bulk material evaluation and is not suit for the observation of the surface of flat samples such as semiconductor devices because the bulk information is mixed. To overcome this problem, a method incorporating laminography has been developed for planar samples and has been successfully utilized to visualize dislocation loops^9^ and slip-band formation at prior mechanical damage^10^ in silicon wafers. However, the spatial resolution remains at 3 μm.

Scanning micro-topography detects the crystallinity from the diffracted X-ray intensity at each irradiation point by scanning the focused X-rays on a sample. A three-dimensional strain image of the region where a basal plane dislocation was converted to a screw dislocation was observed with a spatial resolution of about 1 μm^11, 12^. However, the field of view was limited to 100 μm and required a long measurement time. In short, there is currently no method to evaluate the crystallinity of flat samples such as semiconductor devices in three dimensions with a spatial resolution of 1 μm over a large (more than 1 mm^2^) field of view.

In light of this background, we have developed a novel three-dimensional micro X-ray topography (3D μ-XRT) that combines conventional sectional topography in the Bragg case with focused sheet-shaped X-rays. The depth resolution, depending on the focused X-ray beam size, is expected to reach 1 μm with a large field of view thanks to utilizing a state-of-the-art X-ray focusing system with aspheric total reflection mirrors. In addition, the measurement time can be shortened by using one-dimensional scanning.

## Results

We conducted a feasibility test of the 3D μ-XRT using the monochromatic SR of 1.0 μm and 2 mm in the vertical and horizontal directions, respectively, formed by the focusing system at BL16XU of SPring-8. SiC (2-2010) X-ray diffraction of a 4H-SiC power device chip (3.3-kV double-implanted SiC MOSFET^13^) consisting of a 30-μm epitaxial layer on the surface and 4°-off-cut (0001) was used. The SR energy was set to 10.5 keV, the calculated incident angle (ω) was 10.2 degrees, the exit angle (2θ) was 84.3 degrees, and the X-ray penetration depth was 40 μm. The sample was scanned along the sample surface (Y-axis) with 2.5-μm steps. The number of scanning points was 3,500, each topogram was acquired with an exposure time of 2 s, and the total measurement time was about 2 h.

**Figure 1:**
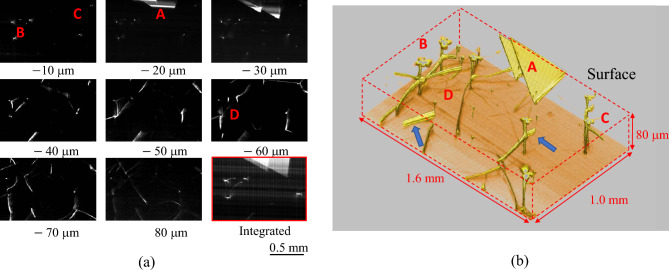
(**a**) Cross-sectional topogram of a SiC chip every 10 μm from the surface and integrated sectional topograms from the surface to 40-μm depth (lower-right). (**b**) 3D topogram.

Figure 1a shows cross-sectional topograms every 10 μm from the surface and Fig. 1b shows a 3D volume rendering topogram of the same area. Stacking faults (A) in the epitaxial layer, basal plane dislocations (BPD) under the epitaxial layer (D), and threading screw (TSD) and threading edge (TED) dislocations (B, C) penetrating the layer are clearly visualized in both figures. The complex connections between the BPD, TSD, and TED, as well as the BPD → SF and BPD → TED conversions^14, 15^, were also clearly observed near the boundary between the epitaxial layer and the substrate. The three-dimensional curvature of the BPD, which provides important thermal information for the fabrication process, can also be clearly visualized. Note that some BPDs have sub-fringes and blurring (blue arrows). This blurring might be caused by the Pendellösung fringes in the Bragg case^16, 17^. The image circled by the red frame in the lower right of Fig. 1a was obtained by integrating sectional topograms from the surface to a depth of 40 μm, which corresponds to a conventional topogram. It is obvious that the above 3D information cannot be obtained from the integrated topogram.

**Figure 2:**
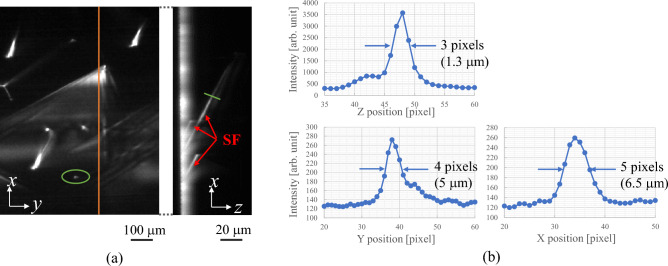
(**a**) Cross-sectional topogram 30 μm below from the surface (left) and sagittal topogram at the orange line (right). (**b**) Intensity line profiles at stacking faults indicated by green line in sagittal topogram (top) and at screw dislocation indicated by green circle in cross-sectional topogram (bottom).

Figure 2a shows a cross-sectional topogram (30 μm below the surface) of different areas of the same sample and a sagittal topogram at the orange line. Note that the z-axis scale of the sagittal topogram was magnified by 5 times to clearly visualize stacking faults and dislocations. The sagittal topogram clearly shows the stacking fault (SF) extending from the surface to the bottom of the epitaxial layer (30 μm).

The upper chart in Fig. 2b shows the line profile of the stacking faults indicated in the green line in the sagittal topogram of (a). The full width at half maximum (FWHM) of the peak is about 3 pixels corresponding to 1.3 μm. Therefore, assuming that the thickness of the stacking faults is negligible, the depth resolution in the 3D μ-XRD is estimated to be 1.3 μm. This value is very close to the resolution expected from the focused beam size (1.0 μm).

The lower charts in Fig. 2b show the intensity line profiles in the X and Y directions for the screw dislocation indicated by the green ellipses in the cross-sectional topogram of (a). The FWHM of the diffraction peak is 6.5 μm and 5 μm in the X and Y directions, respectively. The FWHM is given by a convolution of the blurring of the detection system derived from the beam size and the X-ray imager, and the spatial spread of the screw dislocation, therefore, the actual spatial resolution is expected to be smaller than the obtained FWHMs. The resolution in the X direction is in close agreement with the value calculated from the horizontal divergence angle of the X-ray beam (0.5 mrad) and the camera length (10 mm). The resolution in the Y direction is also in close agreement with the value calculated from the footprint of the X-ray beam on the sample (1/sin(ω) ~ 5.5 μm). The horizontal angular divergence of the focused X-ray beam was estimated to be 1 mrad calculated from the angular range of X-ray diffraction by scanning the sample using the ω table, which is almost the same value expected from the X-ray optical configuration.

By narrowing the vertical aperture of the pinhole and the slit to 5 μm, the vertical angular divergence of the SR irradiating the sample can be reduced to 0.04 mrad (~ 8 arcsecs), although the X-ray intensity is reduced by two orders of magnitude. This divergence angle is almost equivalent to the diffraction width of SiC, and it is expected to make it possible to measure small lattice distortions.

## Discussion

**Figure 3:**
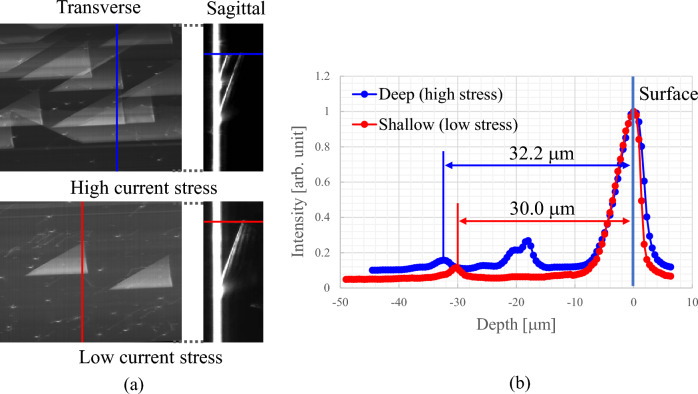
(**a**) Topograms calculated by integrating section topograms from surface to 40-μm depth and (**b**) line profiles at the blue and red lines in sagittal topograms from (**a**). Depth was calculated as 32.2 and 30.0 μm for high and low current stress samples, respectively, using the distance between the two peaks.

We measured two types of SiC device chips with stacking faults (SF) generated by different current stresses (70A/cm^2^ for 0.5 h and 700 A/cm^2^ for 0.5 h) (i.e., different depths) under the same conditions shown in Fig. 1 to determine whether the depth of SF could be evaluated nondestructively and quantitatively by 3D μ-XRT. Figure 3a shows the topogram calculated by integrating section topograms from the surface to the depth of 40 μm (corresponding to the conventional topogram) and the sagittal topogram at the blue and red lines. Figure 3b shows the line profiles of the blue and red lines in the sagittal topogram from Fig. 3a. The depths of the stacking fault were clearly identified and calculated as 33.2 and 30.0 μm for the high and low current stress samples, respectively, using the distance between the two peaks (surface and stacking fault). The depth error was estimated to be 1.3 μm from the depth resolution, which indicates that the depth of stacking faults can be evaluated non-destructively with an accuracy of 1.3 μm. Note that, the SFs are generated not only at the epitaxial interface but also in the deeper region than the interface depending on the current stress^18^.

**Figure 4:**
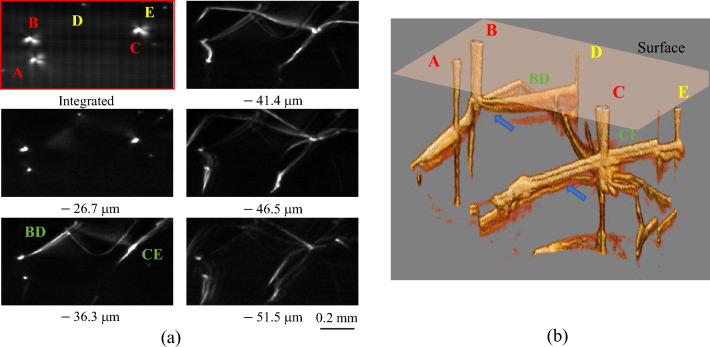
(**a**) Integrated topogram (top left) and cross-sectional topograms at various depths. (**b**) 3D volume-rendering topogram of a region with a mixture of screw and edge dislocations. Both dislocations form a complex network with basal plane dislocations.

We evaluated the distinguishability between threading screw and threading edge dislocations using the same 3D topogram data as that shown in Fig. 2. Figure 4a shows the topogram obtained by integrating sectional topograms from the surface to the depth of 40 μm (upper left) along with cross-sectional topograms at various depths from the surface, and Fig. 4b shows a 3D volume rendering topogram of the same region. The threading screws (A, B, C) and edge dislocations (D, E) can be clearly distinguished in the integrated topogram by the spreading size of the crystal distortion^19^. In addition, both the screw and edge dislocations were intricately connected to the basal plane dislocations and formed a kind of network at depths greater than 36 μm. The edge dislocations D and E are connected to B and C underground (BD and CE), respectively, and D and E are considered mixed dislocations. Note that the TSD generated from TED-TSD conversion would be the TMD mixed with the “a” component of the Burgers vector^20, 21^.

Recently, BPD prismatic slip in SiC crystals larger than 6 inches grown by the physical vapor transport (PVT) method was observed^22^, and a simulation using a thermal model showed that the slip was related to the radial thermal gradient during PVT^23^. Therefore, by comparing the simulation results with the 3D BPD geometry data obtained by 3D μ-XRT, it is expected that the thermal stress during the PVT process will be better analyzed, and the simulation's accuracy will be improved, contributing to the fabrication of high-quality SiC with low distortion.

We assume that the dislocation lines on the basal plane that look double (indicated by blue arrows) are Pendellösung fringes in the Bragg case, the same as that in Fig. 3. We can expect to improve the accuracy of identifying the location and defect type by combining the 3D μ-XRT with simulations based on the Takagi-Taupan equation^24, 25^.

**Figure 5:**
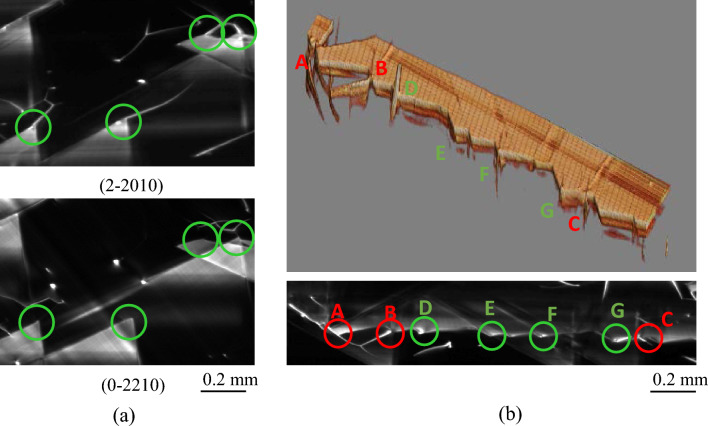
(**a**) Topograms of SiC (2–210) and (0–2210) diffraction integrated from depths of 28–41 μm below the surface. (**b**) 3D volume rendering topogram of the bottom of a stacking defect and topogram integrated from depths of 28–41 μm.

We performed a feasibility test of the evaluation of the Burgers vector direction and the crystallinity of the stacking faults near the boundary between the substrate and epitaxial layer. Figure 5a shows the topograms obtained by integrating the sectional topograms from depths of 28–41 μm acquired using SiC (2-2010) and (0-2210) (in-plane rotation sample angle φ = 30°) diffraction, respectively, under the same conditions as those in Fig. 2. As indicated by the green circle, the upper topogram clearly visualizes the stacking fault tip region connected to the basal plane dislocation, whereas the lower topogram does not show the any connected basal plane dislocation. This result indicates that the orientation of the Burgers vector can be analyzed in three dimensions by using topograms obtained at different angle of in-plane rotation, as in the conventional topography.

Figure 5b shows a 3D volume-rendering topogram of the bottom of the stacking faults and a topogram obtained by integrating sectional topograms from depths of 28–41 μm below the surface. As we can see, the bottom of the stacking fault has a complex structure with a mixture of convex areas connected to the basal plane dislocations (A, B, and C) and unconnected concave areas (D–G). Note that D is a screw dislocation that grazes the bottom of a stacking fault, and is an independent defect from the stacking fault. As shown in this result, 3D μ-XRT enables us to analyze the three-dimensional structure of BPDs, SFs, TSDs, and TEDs, which are difficult to detect by using conventional topography. Our method is thus a powerful tool to elucidate the formation mechanisms underlying these defects and to improve the reliability of SiC power devices.

## Conclusion

In this work, we proposed a novel 3D μ-XRT method that combines Bragg-case section topography with a one-dimensional X-ray beam focused by a total reflection mirror. Feasibility observations of SiC power device chips performed using monochromatic 1-μm-focused SR with an energy of 10.5 keV at BL16XU of SPring-8 in Japan demonstrate that stacking faults, basal plane, screw, and edge dislocations can be successfully visualized in a three-dimensional manner. The depth resolution estimated from the stacking fault size was 1.3 μm. Furthermore, it was possible to conduct a quantitative analysis of the depth of stacking faults and to visualize the three-dimensional network of screw, edge, and basal plane dislocations in the substrate. As a next step, we plan to shorten the measurement time by optimizing the measurement conditions, and to visualize the expansion of stacking faults with increasing current stress (operand topography^13, 26^) in a three-dimensional manner. In addition, we plan to investigate the relationship between detected crystal defects and dislocations and impurities by fluorescence analysis using scanning X-ray fluorescence microscopy rebuilt by re-inserting a second focusing mirror.

## Methods

**Figure 6:**
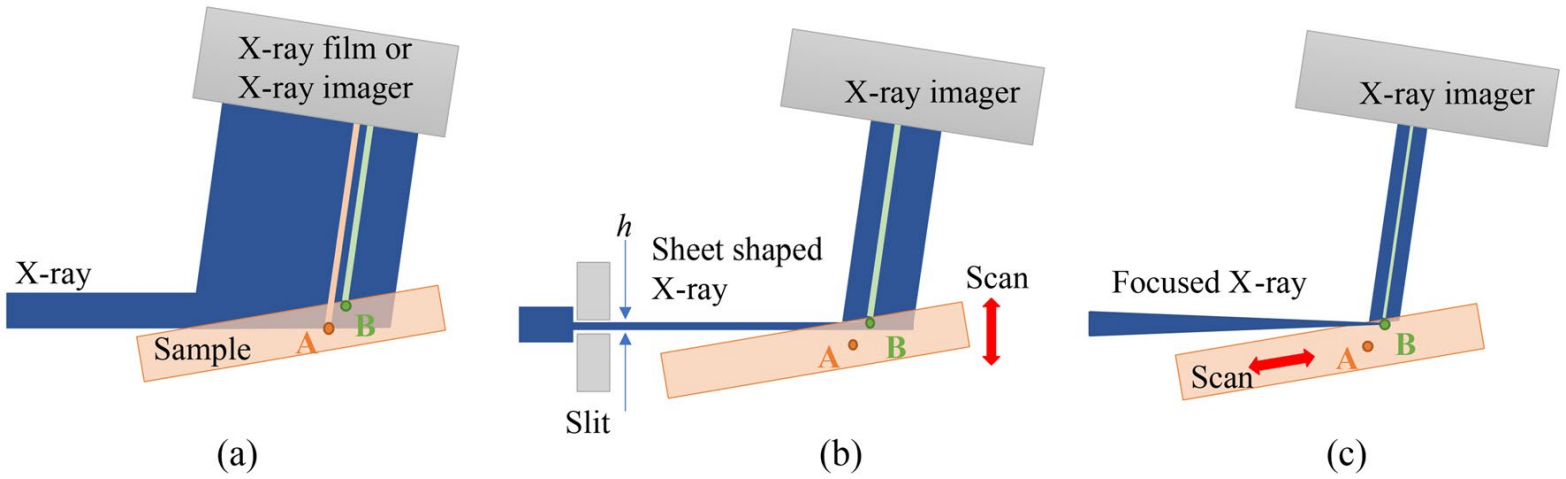
Schematic view of (**a**) conventional reflection topography (Bragg-case), (**b**) reflection section topography, and (**c**) the proposed 3D μ-XRT using a sheet-shaped focused X-ray beam.

X-ray topography detects crystallinity information from X-ray images diffracted by a sample using Bragg or Laue-Case X-ray diffraction, as shown in Fig. 6a. Since the intensity of the diffracted X-rays in each region of the sample depends on the crystal defects and distortions, these defects and distortions can be visualized from the intensity changes. However, the intensity change is integrated along the optical path, which means the depth direction information cannot be obtained. Conventional section topography has therefore been utilized to detect the three-dimensional location of defects and distortions in a sample by means of multiple topograms acquired using sheet-shaped X-rays while scanning the sample, as shown in Fig. 6b.

The depth resolution of the section topography depends mainly on the height *h* of the incident X-ray beam at the irradiated position. The sheet-shaped X-ray beam is usually formed by an X-ray slit with an opening aperture *D*, and the beam height *h'* at the irradiated position on the sample is given by the summation of *D* and the beam broadening due to diffraction. Therefore, *h*' cannot be infinitely small due to the diffraction limit, and the minimum value is theoretically calculated from *D*, the X-ray wavelength λ, and the distance *x* between the slit and the irradiation position. For example, *h*' is calculated as 3 μm for *D* = 1 μm, λ = 0.1 nm, and *x* = 10 mm. In addition, the slit cuts the beam size down to 1/1000 or less, which reduces the X-ray intensity by the same ratio. Thus, even for very strong X-rays such as synchrotron radiation, a long measurement time is required. Another problem is that it is technically quite difficult to make such a narrow slit with an opening aperture of a few microns. In principle, it is impossible for conventional sectional topography to achieve a depth resolution of less than 1 μm.

This limitation can be circumvented by using a sheet-shaped beam focused in one dimension by an X-ray focusing device. The X-ray beam is focused separately in the vertical and horizontal directions by two total reflection mirrors in a Kirkpatrick-Baez (KB) optical configuration. Therefore, one-dimensional focused X-ray is obtained by retracting one mirror from the optical beam path, as shown in Fig. 6c (in this case, the horizontal focusing mirror was retracted). The beam size at the focal point can be focused at less than 1 μm by using the latest X-ray focusing system and mirror, and μ-XRT with a depth resolution below 1 μm is expected to be easily achieved. Although the divergence angle of the X-ray beam will be wider than that of the X-ray beam cut by a slit in conventional section topography, it can be suppressed to less than sub-mrad by utilizing an X-ray slit upstream of the mirror. Note that if the sample is scanned up and down vertically, as in conventional section topography, the focal point and the sample irradiation position will be misaligned. Therefore, the sample has to be scanned parallel to the surface so that the focal point of the X-ray beam and the sample irradiation point always coincide, as shown in Fig. 6c.

**Figure 7:**
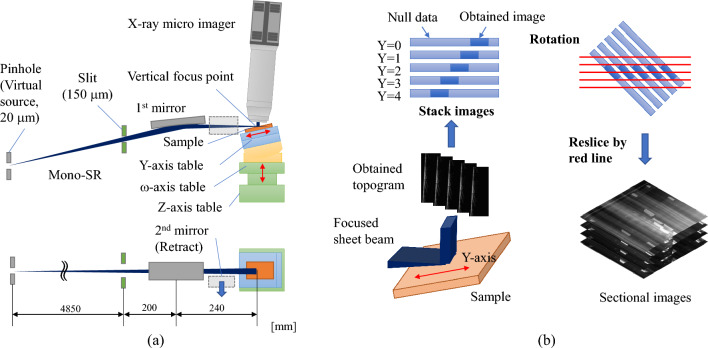
(**a**) Schematic view of 3D μ-XRT system constructed at BL16XU of SPring-8 and (**b**) image processing procedure.

We designed the 3D μ-XRT system using the microbeam system^27^ installed at the beamline BL16XU of the SPring-8 in Japan. As shown in Fig. 7a, the system consists of a focusing mirror, a sample positioner, and an X-ray micro-imager. The white SR emitted from the undulator of the beamline (BL16XU) is monochromated by a double crystal monochromator (DCM) using Si(111) diffraction with energy dispersion (dE/E) of 10^–4^. The SR is next reflected by the bend cylindrical-shaped total reflection pre-mirror to focus on a pinhole (virtual source point of the focusing system). The SR passes through the pinhole is shaped by a slit in front of the focusing mirror and is focused on the sample by the total-reflection elliptical focusing mirror in vertical directions. Note that a second focusing mirror for the horizontal direction is retracted from the optical beam path to form a sheet-shaped X-ray beam in this optical configuration.

The incidence angle of the mirror is 5 mrad, and the distances from the pinhole to the center of the mirrors and from the center of the mirrors to the focal point are 5,050 mm and 240 mm, respectively. Hence, the reduction ratio is approximately 1/20, and the X-ray beam can be focused to 1 μm by setting the pinhole vertical aperture to 20 μm. We also set the vertical aperture of the slit to 150 μm, and reduced the vertical divergence angle of the SR irradiating the sample to 0.6 mrad, which is sufficiently wide compared to the diffraction width of SiC (~ 7 μrad). Therefore, the image contrast was dominated by kinematical diffraction. Each topogram was acquired at the maximum diffraction intensity angle by scanning ω-table.

The sample positioner consists of a swivel table that adjusts the incident angle of the X-ray to satisfy the Bragg diffraction condition, a Y-axis linear table that scans the sample parallel to the sample surface to keep the X-ray focal point and the irradiation point of the sample at the same position, and Z-axis and X-axis tables that adjust the vertical and horizontal positions of the sample. All tables are driven by a stepping motor and controlled remotely.

A lens-coupled X-ray imager (Rad device, Xsight Micron™) is used for the X-ray micro imager. The incident X-rays are converted to visible light by a phosphor and then imaged onto an sCMOS visible camera (Andor Zyla, pixel size: 6.5 μm, 2048 × 2048 pixels) by the visible light lens system. We utilize a 5 × objective lens with the pixel size of 1.3 μm and the field of view of 2.6 mm^2^. The imager is mounted on the 2θ arm of the X-ray diffractometer of the microbeam system. To reduce image blurring, we limit the distance between the X-ray imager and sample to within 5 mm using the height table of the 2θ arm.

Obtained topograms are shifted when scanning the sample along the Y-axis (as shown in Fig. 7b), so we reconstruct a standard 3D topogram with a ratio of 1:1:1 and orthogonally intersect each axis by shifting, stacking, and rotating each topogram using SAKAS-Viewer^28^ and Image J.

## Data Availability

The datasets generated during and/or analyzed during the current study are available from the corresponding author on reasonable request.
